# Motor Rhythm Dissection From the Backward Circuit in *C. elegans*

**DOI:** 10.3389/fnmol.2022.845733

**Published:** 2022-03-16

**Authors:** Bin Yu, Ya Wang, Shangbang Gao

**Affiliations:** ^1^Key Laboratory of Molecular Biophysics of the Ministry of Education, College of Life Science and Technology, Huazhong University of Science and Technology, Wuhan, China; ^2^Department of Geriatrics, Tongji Hospital of Tongji Medical College, Huazhong University of Science and Technology, Wuhan, China

**Keywords:** motor rhythm, rPSCs, ion channels, oscillator, reversal motor circuit, CPG, *C. elegans*

## Abstract

Motor rhythm is initiated and sustained by oscillatory neuronal activity. We recently discovered that the A-class excitatory motor neurons (MNs) (A-MNs) function as intrinsic oscillators. They drive backward locomotion by generating rhythmic postsynaptic currents (rPSCs) in body wall muscles. Molecular underpinning of the rPSCs, however, is not fully elucidated. We report here that there are three types of the rPSC patterns, namely the phasic, tonic, and long-lasting, each with distinct kinetics and channel-dependence. The Na^+^ leak channel is required for all rPSC patterns. The tonic rPSCs exhibit strong dependence on the high-voltage-gated Ca^2+^ channels. Three K^+^ channels, the BK-type Ca^2+^-activated K^+^ channel, Na^+^-activated K^+^ channel, and voltage-gated K^+^ channel (Kv4), primarily inhibit tonic and long-lasting rPSCs with varying degrees and preferences. The elaborate regulation of rPSCs by different channels, through increasing or decreasing the rPSCs frequency and/or charge, correlates with the changes in the reversal velocity for respective channel mutants. The molecular dissection of different A-MNs-rPSC components therefore reveals different mechanisms for multiplex motor rhythm.

## Introduction

Motor rhythm encompasses multiple behaviors that are essential for animal survival, including locomotion, breathing, feeding, and courtship. Motor rhythm is driven by a network of neurons that autonomously generate or sustain oscillatory activities in the absence of external sensory inputs, called central pattern generators (CPGs) (Marder and Bucher, 2001). Locomotor CPGs consist of spinal interneurons (INs) and motor neurons (MNs); they respond to initiation or configuration signals from the central or peripheral nervous systems (Pearson, 1993; Grillner et al., 2008). Depicted as a top-down unidirectional network, the CPG spinal INs instruct the output of MNs to coordinate muscle contractions. MNs in the networks are considered to be merely the relay from pattern-generating circuits to muscles (Grillner, 2006; Kiehn, 2006, 2016). The episodic discharge from locomotor CPG-INs, with particular frequency and phase, ensures precise coordination of muscles controlling the speed and strength of locomotor movements (Wilson, 1961; Grillner, 1975; Wallén and Williams, 1984; Hultborn and Kiehn, 1992; Guertin et al., 1995; Marder and Rehm, 2005; Briggman and Kristan, 2006; Juvin et al., 2007; Ayali and Lange, 2010).

However, electron microscopy reconstructions reveal that MNs make mixed-synapse (chemical and electrical) connections with premotor INs in *C. elegans* (White et al., 1986) and spinal neurons in tadpole larva (Ryan et al., 2016). These physical connections raise the possibility that MNs are not simple “final common path,” but could be considered as an integral part of the locomotor CPGs. Indeed, evoked MN activity retrogradely regulated the activity of CPG-INs or premotor INs was reported across phyla, from the tadpole (Roberts and Perrins, 1995), zebrafish (Song et al., 2016), chick (Wenner and O’Donovan, 1999, 2001), and rodent (Mentis et al., 2005), to leech (Rela and Szczupak, 2003; Szczupak, 2014) and *C. elegans* (Liu et al., 2017). In addition, disruption of the MN activity affects the upstream CPG-IN patterns and motor rhythm in the crayfish and Drosophila (Heitler, 1978; Matsunaga et al., 2017).

**FIGURE 1:**
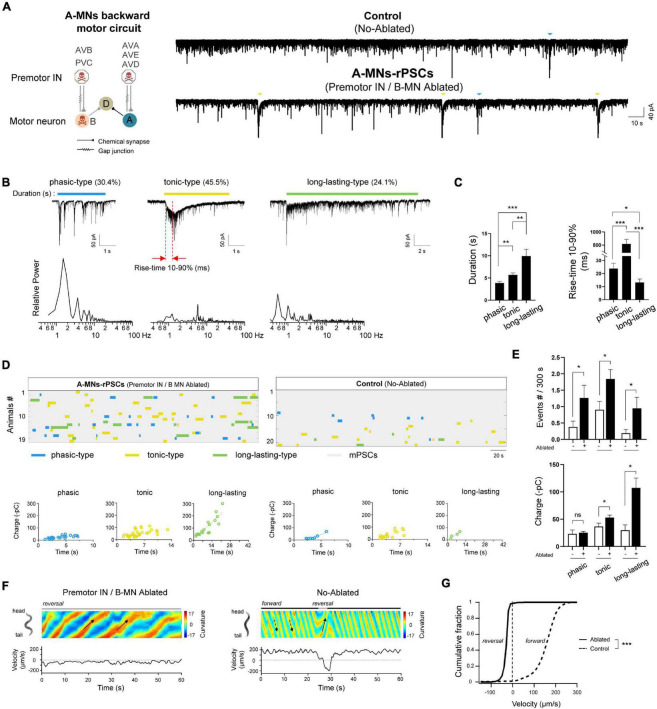
Three types of compound rPSCs constitute A-MNs oscillators. **(A)** *Left*, schematics of the motor circuit components and connectivity. Upon ablation of premotor INs and B-MNs using miniSOG transgene animals, A-MN backward motor circuit, containing A-MNs and D-MNs, was isolated. *Right*, representative spontaneous PSCs trace and continue charge frequency analysis (–pC/3s) from wild-type neuromuscular junction upon the coablation of premotor INs and B-MNs. Rhythmic PSCs events with large charges were recorded. Muscle cell was held at –60 mV. **(B)** Top: three typical compound rPSCs exhibit distinct activation dynamics, including phasic-type, slow activated with tonic spillover PSCs (tonic-type), and fast activated long-lasting-type PSCs (long-lasting-type), respectively. Bottom: frequency spectrum analysis of above rPSCs. **(C)** Quantification of the duration and rise time (10–90%) of different types of events. **(D)** Colormap and charge efficiency (by linear fitting) of all rPSC events from the animals with (Ablated) and without (Control) ablation. Blue dots denote phasic-type events, yellow dots denote tonic-type events, and green dots denote long-lasting-type events. **(E)** Quantification of the frequency and charge of different rPSCs without (−) or with (+) premotor INs and B-MNs ablation. *n* ≥ 19 animals in each group. **(F)** Representative curvature kymogram (*up*) and instantaneous velocity (*bottom*) of free-behaving animals, with (Ablated) and without (Control) ablation. Ablated animals exhibit persistent reversal, as posterior to anterior propagating body bends (arrows). **(G)** Distribution of instantaneous velocity of animals with (Ablated) and without (Control) ablation. The velocity fraction is drastically left-shifted after ablation. *n* ≥ 10 in each group. ***p < 0.001 against non-ablated Control groups by the Kolmogorov–Smirnov test. All data are expressed as means ± SEM. Student’s *t*-test was used **(B,C,E,F)**. Statistical significance is indicated as follows: ns, not significant, *p < 0.05, **p < 0.01, ***p < 0.001 in comparison with that as denoted.

We and others previously discovered that *C. elegans* ventral cord motor circuit contains multiple CPG-like locomotor networks or oscillators for body undulation (Kato et al., 2015; Fouad et al., 2018; Gao et al., 2018; Xu et al., 2018; Wen et al., 2018). They were located in two subcircuits with anticorrelated activity dynamics: premotor INs (AVB and PVC) and B-class MNs (B-MNs) composed forward circuit, and premotor INs (AVA AVE and AVD) and A-MNs composed backward circuit (Figure 1A). Both subcircuits are connected to each other through both electrical and chemical synapses (Chalfie et al., 1985; White et al., 1986). Antagonistic activity between the two subcircuits underlies transition between the directional motor states (Kawano et al., 2011; Kato et al., 2015; Roberts et al., 2016). More importantly, we recently found that, without all premotor INs, the excitatory A-MNs themselves constitute a CPG-like distributed network of oscillators (Gao et al., 2018). The isolated A-MN oscillators drive continuous rhythmic backward locomotion without INs or sensory inputs. These results indicate that A-MNs function as a chain of phase-coupled local oscillators to organize and execute backward locomotion. Therefore, A-MNs are not only the backward movement execution unit, but also a core module of locomotor CPG.

In this study, we address how A-MN’s oscillatory properties are constituted at the physiological and molecular level. We applied dissected *C. elegans* NMJ preparation (Richmond and Jorgensen, 1999; Mellem et al., 2008; Gao and Zhen, 2011; Liu et al., 2013) to record the postsynaptic currents (PSCs) in anterior ventral body muscles to analyze currents that constitute A-MN’s endogenous activity. Previously, we have shown a standard muscle PSCs recording consists of miniature PSCs (mPSCs) and rare patterned events, called rhythmic PSC (rPSCs) bursts that are activated by premotor INs (Gao et al., 2015). These rPSCs are A-MN-dependent and become prominent in premotor INs and B-MNs ablated animals (Gao et al., 2018). Importantly, the frequency and charge of these rPSCs, but not the frequency or amplitude of the mPSC, correlated with the reversal velocity (Gao et al., 2018).

Here, we report three classes of the rPSC, each with distinct kinetics (frequency spectrum, activation rate, and duration) generated by the isolated *C. elegans* backward circuit. These rPSCs constitute the A-MN’s oscillatory property and instruct sustained reversal. By further comparison of the rPSC’s frequency and charge between wild-type and various channel mutant animals, we identified several Ca^2+^, Na^+^, and K^+^ channels that differentially affect each of the three patterns and reversal movement. Our results denoted multiple forms and regulations of *C. elegans* locomotory oscillators.

## Results

### Isolated Backward Motor Circuit Preparation Exhibits Three Types of Rhythmic Postsynaptic Currents

A-class excitatory motor neurons generate spontaneous periodic electric activities independent of premotor INs (Gao et al., 2018). To address how these rhythmic patterns of activity are constituted, we optogenetically isolated a pure A-MN backward motor circuit. A transgenic strain by simultaneous expression of a flavoprotein miniSOG into all premotor INs and B-MNs was used (section “Materials and Methods”). MiniSOG releases singlet oxygen by blue-light illumination that induces acute functional loss and subsequent death of neurons (Qi et al., 2012). Upon ablation, the entire population of all premotor INs and B-MNs were removed, and thus, A-MN backward motor circuit was isolated (Figure 1A). We then recorded spontaneous rPSCs for more than 5 min to collect rhythmic events from the dissected neuromuscular preparation. Strong periodic compound PSCs were observed at all ablated preparations with an average frequency of ∼4 events per 300 s (*n* = 19). These rPSC events were obviously distinct from the mPSCs due to the 5-to 10-fold charge transfer difference (rPSCs 67.50 ± 12.11 vs. mPSCs 7.42 ± 0.41 –pC/3s) (Figure 1A). By contrast, only sporadic rPSC events were observed in non-ablated wild-type preparations with dramatically low frequency (1.52 ± 0.34 events per 300 s) and charge transfer (33.49 ± 4.46 –pC) (*n* = 21) (Supplementary Figures 1A,B). These results clarified that isolated A-MN motor circuits exhibit robust rhythmic PSCs.

To analyze these rPSCs, we reexamined their components. From these pooled traces, at least three types of rPSCs were recorded, with different activation dynamics. The phasic rPSCs consisted of a group of rapid burst depolarizing currents of –50 to –300 pA: each had the burst frequency between 1 and 4 Hz and lasted 3.88 ± 0.36 s (Figures 1B,C). Different from the phasic-type, tonic rPSCs had the highest proportion (45.5%) and slowest activation kinetics: 10–90% rise time 817.98 ± 68.79 ms and a slightly longer duration (5.60 ± 0.45 s). Long-lasting rPSCs had a duration of 9.94 ± 1.53 s and were usually accompanied with a fast initiation current with 10–90% rise time of 13.20 ± 2.61 ms (Figures 1B,C). The tonic and long-lasting types of rPSCs were usually containing high-frequency mPSCs and irregular bursts. Transient PSC bursts or multiphasic mPSCs were also observed sporadically but without predictable rhythmicity (data not shown). Three types of rPSCs are proposed excitatory, since periodic action potential bursts corresponded to these rPSCs after premotor INs and B-MNs were ablated (Gao et al., 2018). Therefore, we isolated various rhythmic electrical signals that undergo the A-MN oscillating activities.

Except for the phasic rPSCs, which are potentiated by activation of premotor INs AVA (Gao et al., 2015, 2018), the characterizations of tonic and long-lasting types of rPSCs have not been reported. To determine how these rPSC components constitute the rhythmic patterns of activity, we pooled all rPSCs events together from all ablated wild-type animals (Figure 1D). In individual preparation, tonic rPSCs occurred either alone or together with others, arising mixed types of rPSCs. The average frequency of each rPSCs is approximately 1–2 events per 300 s, building a total frequency of 3.83 ± 0.58 events per 300 s (Figure 1E and Supplementary Figure 1B). Each type of rPSCs also exhibited different charge transfer, from minimal phasic-type (25.46 ± 2.17 –pC) to maximal long-lasting type (107.27 ± 17.92 –pC), correlated with the rPSC duration (Figures 1D,E and Supplementary Figure 1B). Thus, these rPSCs orchestrated the rhythmic patterns of activity of the A-MN oscillator from the isolated backward motor circuit.

Spontaneous rPSCs were also observed in the same non-ablated transgenic worms without blue-light illumination. By pooling the sporadic rPSC events from 21 non-ablated wild-type (Control) preparations, we noticed that three types of rPSCs were also recorded (Figure 1D). The frequency of each type of rPSCs, however, was significantly lower than that of ablated animals (Figure 1E), which leads to decreased total frequency (Supplementary Figure 1B). The charge of tonic and long-lasting types of rPSCs in these animals was also less than that of ablated worms, which is largely due to shortened duration (Figure 1E and Supplementary Figure 1B). Increased frequency and charge transfer of the rPSCs from the isolated backward motor circuit reflected boosted rhythmic activity of the A-MN oscillator. Taken together, these results demonstrated that rhythmic patterns of activity from A-MNs were constituted by three key components of rPSCs with different activation dynamics.

### The Rhythmic Postsynaptic Currents Promote Backward Movement Propensity and Velocity

To address whether these types of rPSCs from A-MNs are intrinsically functional, we then examined the behavioral response by the ablation of all premotor INs and B-MNs. Consistently, upon ablation, animals with isolated A-MNs exhibited continuous backward movements with periodic stalled by the forward-promoting head oscillations (Figure 1F). The animals without ablation (Control), however, exhibited predominantly forward locomotion with occasional backward interruption, similar to the non-transgenic wild-type N2 strain (data not shown). Consequently, ablation induced a strong left-shift in the velocity curve (Figure 1G). Considering the increased frequency or charge of rPSCs from these ablated animals, our results suggest that these rPSCs provide the intrinsic depolarizing signals that drive coordinated muscle contraction, thus promoting the rhythmic backward movements.

### A Na^+^ Leak Channel NCA/NALCN Is Required for All Rhythmic Postsynaptic Currents

We and others have identified that the sodium (Na^+^) leak channel NCA (Humphrey et al., 2007; Jospin et al., 2007; Yeh et al., 2008; Xie et al., 2013; Flourakis et al., 2015), homolog of the mammalian NALCN (Lu et al., 2007), is critical for rhythmic locomotion. This channel, encoded by two functionally redundant pore-forming subunits *nca-1* and *nca-2*, is expressed ubiquitously in the nervous system and sustains motor activity (Xie et al., 2013; Gao et al., 2015). To identify NCA’s involvement in the backward motor circuit, we examined the motor behaviors of *nca(lf)* mutants with the isolated backward motor circuit preparation.

**FIGURE 2:**
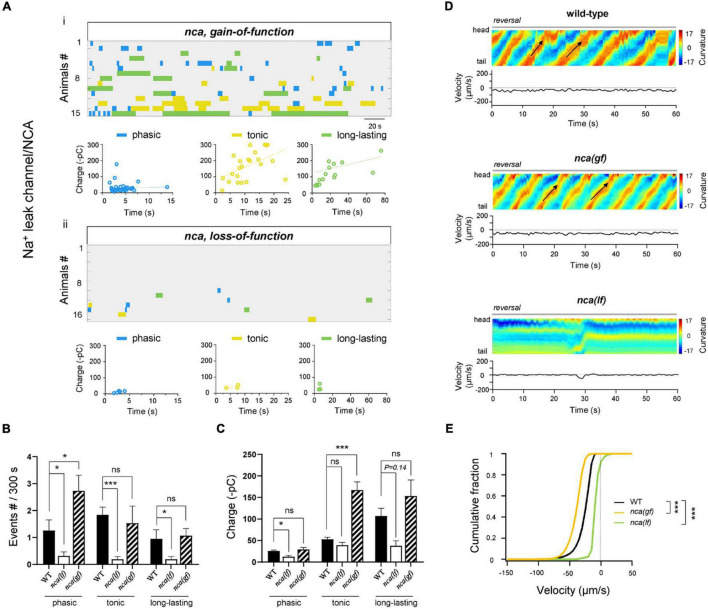
NCA Na^+^ leak channel is required for all types of rPSCs. **(A)** Colormap and charge efficiency (linear fitting) of all rPSC events recorded from ablated *nca-1(hp102; gf)* and *nca-1(gk9; lf); nca-2(gk5; lf)* mutant animals, respectively. Blue dots denote phasic-type events, yellow dots denote tonic-type events, and green dots denote long-lasting-type events. **(B,C)** Quantification of the frequency and charge of different rPSCs in respective genotypes. *n* ≥ 15 animals in each group. **(D)** Representative curvature kymogram (*up*) and instantaneous velocity (*bottom*) of free-behaving animals of respective genotypes after ablation. *nca-1(hp102; gf)* mutant animals exhibit increased reversal, as posterior to anterior propagating body bends (arrows), and *nca-1(gk9; lf); nca-2(gk5; lf)* mutants exhibit stalled backward. **(E)** Distribution of instantaneous velocity of wild-type, *nca-1(hp102; gf)* and *nca-1(gk9; lf); nca-2(gk5; lf)* mutant animals. The velocity fraction is left-shifted in the *nca-1(hp102; gf)* mutant, and right-shifted in the *nca-1(gk9; lf); nca-2(gk5; lf)* mutant, respectively. *n* ≥ 10 in each group. ***p < 0.001 against ablated wild-type by the Kolmogorov–Smirnov test. All data are expressed as means ± SEM. Student’s *t*-test was used **(B,C)**. Statistical significance is indicated as follows: ns, not significant, *p < 0.05, ***p < 0.001 in comparison with that as denoted.

Upon the removal of premotor INs and B-MNs, the reversal velocity of *nca(lf)* mutant animals was decreased when compared to that of wild-type animals (Figures 2D,E). We then asked whether NCA channels regulate the A-MN rPSCs. In *nca(lf)* animals, the phasic rPSCs exhibit a decrease in both frequency and charge. Both tonic and long-lasting rPSCs also exhibit reduced frequency; their charge exhibited the trend of decrease, but failed to reach statistical significance (Figures 2A–C). These results suggest that the NCA Na^+^ leak channel is involved in all rPSCs.

A *gain-of-function* mutation allele of *nca-1* (*nca(gf)*) led to enhanced motor activity (Yeh et al., 2008). We found that in the *nca(gf)* isolated reversal motor circuit, the frequency of phasic rPSCs and charge of tonic rPSCs were significantly increased (Figures 2A–C). Consistently, after the removal of premotor INs and B-MNs, *nca(gf)* mutants exhibited increased reversal velocity when compared to that of wild-type animals. The increased frequency of phasic rPSCs in *nca(gf)* and decreased frequency of all rPSCs in *nca(lf)* mutants indicated that the Na^+^ leak channel has widespread effects on all types of rPSCs.

### Regulation of Tonic and Phasic Rhythmic Postsynaptic Currents by the High-Voltage P/Q/N-Type VGCC

**FIGURE 3:**
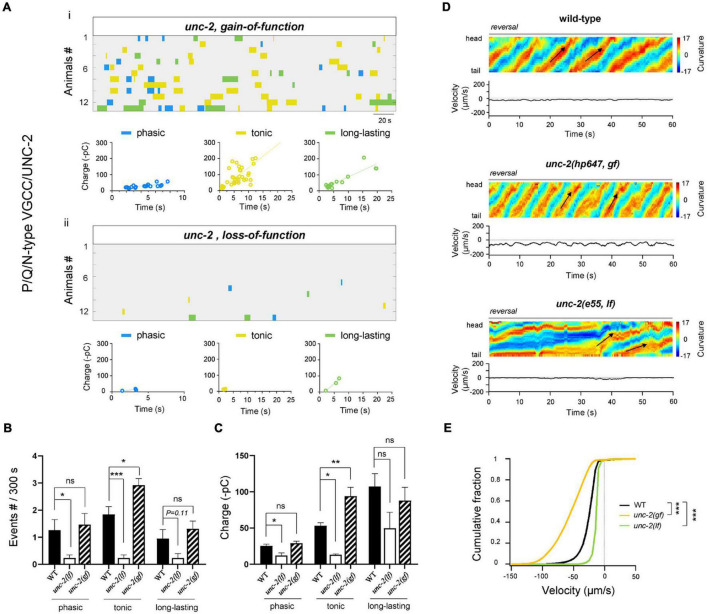
High-voltage P/Q/N-VGCC(UNC-2) facilitates tonic and phasic rPSCs. **(A)** Colormap and charge efficiency (linear fitting) of all rPSC events from *unc-2(hp647; gf)* (*up*) and *unc-2(e55; lf)* (*bottom*) mutant animals, after the coablation of premotor INs and B-MNs. Blue dots denote phasic-type events, yellow dots denote tonic-type events, and green dots denote long-lasting-type events. **(B,C)** Quantification of the frequency and charge of different rPSCs in respective genotypes. *n* = 13 animals in each group. **(D)** Representative curvature kymogram (*up*) and instantaneous velocity (*bottom*) of free-behaving animals of respective genotypes after the ablation. *unc-2(hp647; gf)* mutant animals exhibit persistent fast reversal, as posterior to anterior propagating body bends (arrows), while *unc-2(e55; lf)* mutants exhibit almost sluggish backward locomotion. **(E)** Distribution of instantaneous reversal velocity of wild-type, *unc-2(hp647; gf)* and *unc-2(e55; lf)* mutant animals. The velocity fraction is drastically left-shifted in *unc-2(hp647; gf)*, and right-shifted in *unc-2(e55; lf)* mutants, respectively. *n* ≥ 10 animals in each group. ***p < 0.001 against ablated wild-type by the Kolmogorov–Smirnov test. All data are expressed as means ± SEM. Student’s *t*-test was used **(B,C)**. Kolmogorov–Smirnov test was used **(E)**. Statistical significance is indicated as follows: ns, not significant, *p < 0.05, **p < 0.01, ***p < 0.001 in comparison with that as denoted.

Functional rPSCs are initiated by the periodic neuronal activity, which is established by voltage-gated ion channels (Grillner, 2003), voltage-gated Ca^2+^, Na^+^ channels that provide the excitatory currents for depolarizing the membrane potential, and voltage-gated K^+^ channels that instruct the membrane hyperpolarized currents (Harris-Warrick, 2002). To address the molecular regulators that underlie the above different types of rPSCs, we focused on channel proteins and analyzed the rPSCs frequency or charge in mutants by decreasing and/or increasing the channel activity. All mutant animals were recorded without premotor INs and B-MNs after ablation. High-voltage-activated P/Q/N-type calcium channel (VGCC) is substantially required for coordinated locomotion and Ca^2+^ oscillation of MNs (Büschges et al., 2000; Gao et al., 2018; Huang et al., 2019). In a partial *lf* mutant for the *α-*subunit of the P/Q/N-CaV2α UNC-2, the frequency of tonic and phasic rPSCs was significantly decreased (Figures 3Aii,B), which results in a drastically reduced total frequency of rPSCs (Supplementary Figures 1C,D). The charge transfer of these two types of rPSCs also exhibited a drastic decrease in the *unc-2(lf)* mutant when compared to wild-type animals (Figures 3Aii,C). Neither frequency nor charge transfer of long-lasting rPSCs was affected in this mutant. These results confirm the essential requirement of P/Q/N-type VGCC for A-MN rhythmic oscillations and demonstrate specific regulation of P/Q/N-type VGCC for tonic and phasic types of rPSCs.

Altered A-MN activity corresponded with changes in reversal velocity. We then asked whether the reduced tonic and phasic types of rPSCs in *unc-2(lf)* had any behavioral consequences. Upon the ablation of premotor INs and B-MNs, *unc-2(lf)* mutants exhibited strikingly reduced reversal velocities than ablated wild-type animals (Figures 3D,E), which reinforce the requirement of P/Q/N-type VGCC for locomotion. This result indicates that activity change in two types of rPSCs affects locomotion substantially. More interesting, we further examined the effect of a gain-of-function (*gf*) mutation in *unc-2* with prolonged channel opening kinetics (Huang et al., 2019). In contrast to the case of *unc-2(lf)* mutants, upon ablation, only the tonic rPSCs in the *unc-2(gf)* mutant exhibited increased frequency and charge (Figures 3A–C). Intriguingly, ablated *unc-2(gf)* mutants also exhibited dramatically increased reversal velocities than ablated wild-type animals (Figures 3D,E), which suggests a dominant effect of tonic rPSCs on locomotion.

Collectively, through decreasing or increasing the activity of P/Q/N-type VGCC/UNC-2, these results demonstrate the differential regulation of tonic and/or phasic rPSCs by the high-voltage-activated P/Q/N-type VGCC is sufficient to alter the property of reversal movements.

### The Requirement of High-Voltage L-Type High-Voltage-Activated P/Q/N-Type Calcium Channel for the Tonic Rhythmic Postsynaptic Currents

**FIGURE 4:**
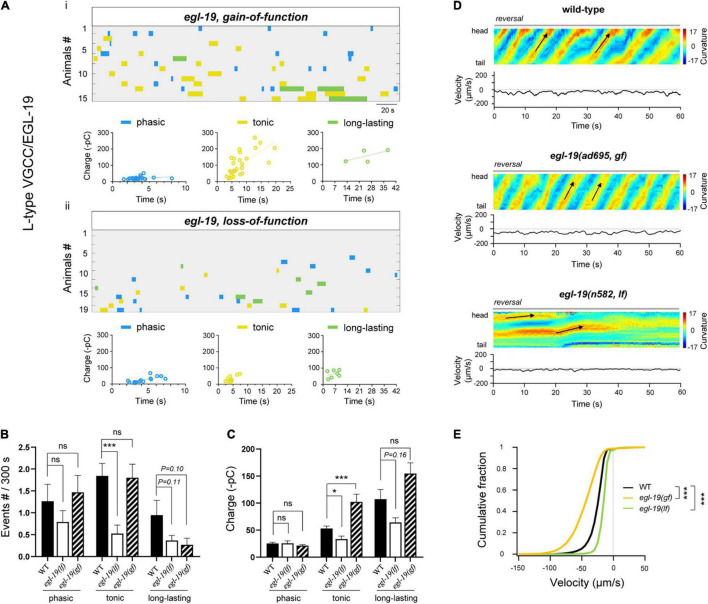
High-voltage L-VGCC (EGl-19) specifically regulates tonic-rPSCs. **(A)** Colormap and charge efficiency (linear fitting) of all rPSC events from *egl-19(ad695; gf)* and *egl-19(n582; lf)* mutant animals, after the coablation of premotor INs and B-MNs. Blue dots denote phasic-type events, yellow dots denote tonic-type events, and green dots denote long-lasting-type events. **(B,C)** Quantification of the frequency and charge of different rPSCs in respective genotypes with premotor INs and B-MNs ablation. *n* ≥ 16 animals in each group. **(D)** Representative curvature kymogram (*up*) and instantaneous velocity (*bottom*) of free-behaving animals of respective genotypes after ablation. e*gl-19(ad695; gf)* mutants exhibit increased reversal, as posterior to anterior propagating body bends (arrows). *egl-19(n582; lf)* mutants exhibit reduced backward locomotion. **(E)** Distribution of instantaneous velocity of wild-type, e*gl-19(ad695; gf)* and *egl-19(n582; lf)* mutants. The velocity fraction is left-shifted in e*gl-19(ad695; gf)*, and right-shifted in *egl-19(n582; lf)* mutants, respectively. *n* ≥ 10 in each group. ***p < 0.001 against ablated wild-type by the Kolmogorov–Smirnov test. All data are expressed as means ± SEM. Student’s *t*-test was used **(B,C)**. Statistical significance is indicated as follows: ns, not significant, *p < 0.05, ***p < 0.001 in comparison with that as denoted.

Multiple types of voltage-activated calcium currents are also recorded in the locomotor network neurons (Tegnér et al., 1997; Guertin and Hounsgaard, 1998). *C. elegans* genome has two more VGCC genes, namely, *egl-19*, which encodes L-type VGCC and *cca-1* that encodes T-type VGCC, respectively. Both of them were reported to be expressed in the ventral nerve cords (Lee et al., 1997; Bargmann, 1998; Consortium, 1998). To identify whether they are also involved in rPSC regulation, we examined the *lf* mutants of the genes. In mutants containing a partial *lf* allele for the pore-forming α*-*subunit of the L-VGCC CaV1α EGL-19, tonic rPSCs were significantly decreased, in both frequency and charge, when compared to wild-type animals (Figures 4A–C and Supplementary Figures 1C–E; *egl-19(n582; lf)*). The reversal velocity in ablated *egl-19(lf)* mutants was also substantially attenuated (Figures 4D,E). By contrast, in a *lf* allele mutant of the T-type VGCC CaV1α CCA-1, all types of rPSCs were readily recorded [Supplementary Figures 1C–E, 2A–C; *cca-1(ad1650; lf)*]. The reversal velocity was also unaltered in *cca-1(lf)* mutants (Supplementary Figures 2D,E). These results demonstrate that L-VGCC/EGL-19, but not T-VGCC/CCA-1, is critical for rPSC regulation, especially for tonic ones.

According to the results that the *unc-2(gf)* mutant exhibited counter-effects on rPSCs compared to the *unc-2(lf)* mutant, we predicted that a *gf* mutant of *egl-19* would show improved rPSCs. Indeed, *egl-19(ad695)*, a *gf* mutant in which the EGL-19 I-V curve is left-shifted, inducing channel activation at more negative potentials (Lainé et al., 2014), exhibited obviously increased tonic rPSCs in charge (Figures 4Ai,C). Interestingly, *egl-19(gf)* mutants also exhibited significantly increased reversal velocity than ablated wild-type animals (Figures 4D,E), which confirms the importance of tonic rPSCs. No change in rPSCs frequency in the *egl-19(gf)* mutants suggests that increased charge only is sufficient to improve movement.

### Ca^2+^-Activated K^+^ Channel SLO-1 Inhibits the Tonic Rhythmic Postsynaptic Currents

**FIGURE 5:**
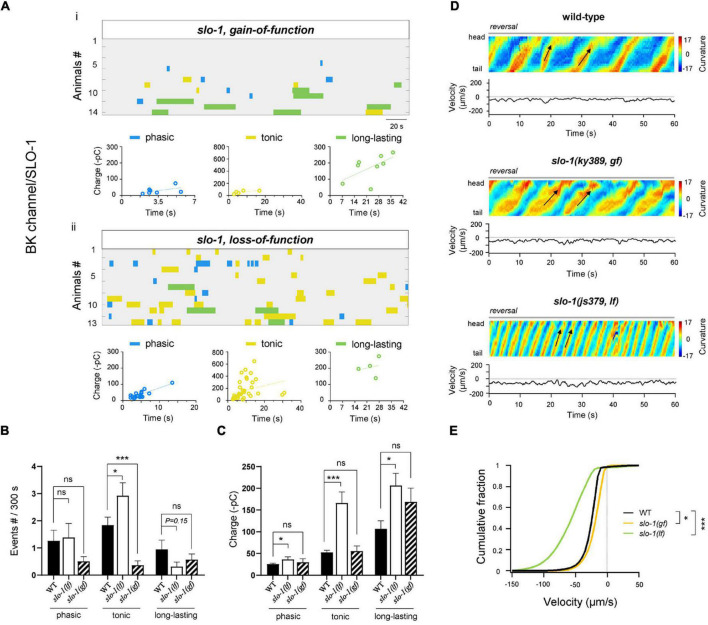
BK/SLO-1 channel inhibits tonic- and phasic-type rPSCs. **(A)** Colormap and charge efficiency (linear fitting) of all rPSC events recorded from ablated *slo-1(ky389; gf)* and *slo-1(js379; lf)* mutant animals, respectively. Blue dots denote phasic-type events, yellow dots denote tonic-type events, and green dots denote long-lasting-type events. **(B,C)** Quantification of the frequency and charge of different rPSCs in respective genotypes with premotor INs and B-MNs ablation. *n* ≥ 14 animals in each group. **(D)** Representative curvature kymogram (*up*) and instantaneous velocity (*bottom*) of free-behaving animals of respective genotypes. *slo-1(js379; lf)* mutant animals exhibit faster reversal, as posterior to anterior propagating body bends (arrows). **(E)** Distribution of instantaneous velocity of wild-type, *slo-1(ky389; gf)*, and *slo-1(js379; lf)* mutant animals. The velocity fraction is drastically left-shifted in *slo-1(js379; lf)* mutants, and right-shifted in *slo-1(ky389; gf)* mutant animals. *n* ≥ 10 animals in each group. *p < 0.05, ***p < 0.001 against ablated wild-type by the Kolmogorov–Smirnov test. All data are expressed as means ± SEM. Student’s *t*-test was used **(B,C)**. Statistical significance is indicated as follows: ns, not significant, *p < 0.05, ***p < 0.001 in comparison with that as denoted.

Potassium (K^+^) channels regulate the repolarization of membrane potentials, thus inhibiting the neuronal activity. Ca^2+^-activated BK-type K^+^ channel, activated by Ca^2+^ influx through P/Q/N-type VGCC, modulates afterhyperpolarization of spinal neurons, which is required for locomotion (Wikström and El Manira, 1998). We reasoned that the BK channel is also important for A-MNs-CPG. First, SLO-1, the *C. elegans* BK-type K^+^ channel, has expression in MNs (Wang et al., 2001). Second, upon the ablation of premotor INs and B-MNs, the frequency and charge of tonic rPSCs were significantly increased in *slo-1(lf)* mutants compared to wild-type animals (Figures 5Aii,B). The charge of phasic and long-lasting rPSCs was also increased (Figure 5C), which indicates that the BK/SLO-1 channel has diverse regulations of rPSCs. Furthermore, *slo-1(lf)* mutant animals exhibited dramatically increased reversal velocity after the removal of premotor INs and B-MNs (Figures 5D,E). These results denote that BK/SLO-1 channel is indeed required for the A-MN rPSCs.

We also tested the rPSCs and reversal velocity in a *gf* mutant of *slo-1*, which exhibited sluggish locomotion (Troemel et al., 1999). Upon ablation, the frequency of tonic rPSCs was significantly decreased (Figures 5Ai,B). The charge of all types of rPSCs was, however, comparable to the ablated wild-type. Behaviorally, the reversal velocity was modestly inhibited in *slo-1(gf)* mutants (Figures 5D,E). Taken together, our results indicate that BK/SLO-1 channel, such as NCA channel, has wide effects on the rPSCs.

### Two Kv Channels Modestly Reduce Some Rhythmic Postsynaptic Currents Components

**FIGURE 6:**
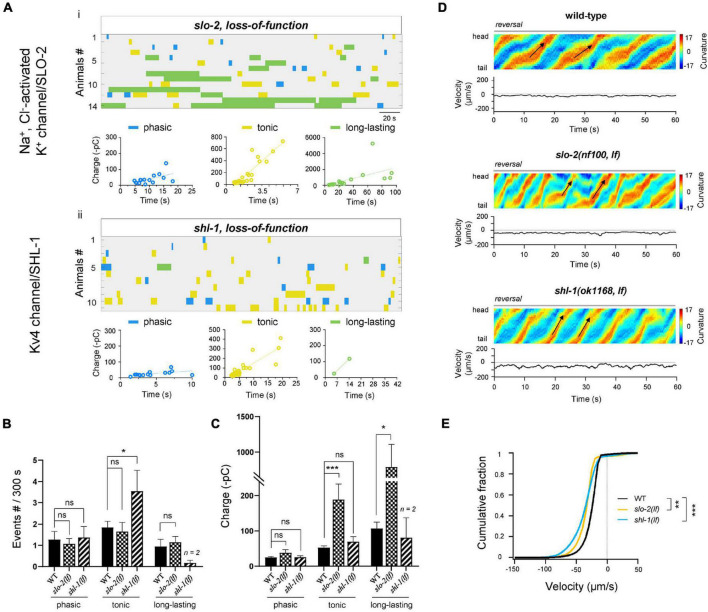
K_Na_/SLO-2 and Kv4/SHL-1 channels differentially regulate tonic- and long-lasting-type rPSCs. **(A)** Colormap and charge efficiency (linear fitting) of all rPSC events recorded from ablated *slo-2(nf100; lf)* and *shl-1(ok1168; lf)* mutant animals, respectively. Blue dots denote phasic-type events, yellow dots denote tonic-type events, and green dots denote long-lasting-type events. **(B,C)** Quantification of the frequency and charge of different rPSCs in respective genotypes. *n* ≥ 11 animals in each group. Only 2 long-lasting-type rPSCs events were recorded in *shl-1(lf)* mutants. **(D)** Representative curvature kymogram (*up*) and instantaneous velocity (*bottom*) of free-behaving animals of respective genotypes. Ablated *slo-2(nf100; lf)* and *shl-1(ok1168; lf)* animals exhibit increased reversal, as posterior to anterior propagating body bends (arrows). **(E)** Distribution of instantaneous velocity of wild-type, *slo-2(nf100; lf)*, and *shl-1(ok1168; lf)* mutant animals. The velocity fraction is drastically left-shifted both in *slo-2(nf100; lf)* and in *shl-1(ok1168; lf)* mutant animals. *n* ≥ 10 in each group. **P < 0.01, ***p < 0.001 against ablated wild-type by the Kolmogorov–Smirnov test. All data are expressed as means ± SEM. Student’s *t*-test was used **(B,C)**. Statistical significance is indicated as follows: ns, not significant, *p < 0.05, ***p < 0.001 in comparison with that as denoted.

Except of BK/SLO-1 channel, *C. elegans* genome predicts > 70 K^+^ channel components (Bargmann, 1998). Three of them, *slo-2*, *shl-1*, and *shk-1*, were reported to be expressed in MNs (Yuan et al., 2000; Fawcett et al., 2006). *Slo-2* encodes a subunit of a Na^+^ and Cl^–^-activated K^+^ (K_Na_) channel, whereas *shk-1* and *shl-1* are α-subunits of the Kv1 and Kv4 voltage-gated K^+^ channels, respectively. Among these mutants, only *kv4/shl-1(lf)* exhibited ∼2-fold frequency increase of tonic rPSCs. By contrast, *k_*Na*_/slo-2(lf)* and *kv1/shk-1(lf)* have no effects on the frequency at all types of rPSCs (Figures 6A,B and Supplementary Figures 3A,B). On the other hand, when compared to ablated wild-type, both *k_*Na*_/slo-2(lf)* and *kv1/shk-1(lf)* increased the charge. Specifically, *k_*Na*_/slo-2(lf)* displayed ∼10-fold and ∼4-fold charge increase of long-lasting and tonic rPSCs, respectively (Figure 6C). *Kv1/shk-1(lf)* displayed a moderately charge increase only in tonic-type (Supplementary Figure 3C). Behaviorally, the reversal velocity was significantly increased in *k_*Na*_/slo-2(lf)* and *kv4/shl-1(lf)* (Figures 6D,E). The moderate charge increase of tonic rPSCs in *kv1/shk-1(lf)* mutant, however, had no obvious effect on reversal velocity (Supplementary Figures 3D,E). Thus, these results suggest that the K_Na_/SLO-2 channel is required for regulating tonic and long-lasting rPSCs, whereas Kv4/SHL-1 channel is specific for tonic-type.

## Discussion

Recent studies uncover that in the compact *C. elegans* motor system, excitatory ventral cord MNs are the rhythm generators for body movement (Fouad et al., 2018; Gao et al., 2018; Xu et al., 2018). The A-type MNs, as backward oscillators, exhibit periodic electrical activities that regulate rhythmic reversal movement (Gao et al., 2018). However, how the A-MN’s oscillatory properties are constituted and the molecular identities of underlying channel conductance remain elusive. Here, we report multiple types of A-MN rPSCs. Genetic disruption of specific types of rPSCs substantially affects the reversal velocity; thus, all types of rPSCs likely contribute to reversal movement. This is the first report of multiple types of rhythmic electrical activities by the same MN circuit in a *C. elegans* preparation.

### The Origin of Heterogeneous Rhythmic Postsynaptic Currents

Oscillatory motor CPG activities drive repetitive movement tracks (Marder and Rehm, 2005; Grillner, 2006). Our observation shows that the same circuit can generate heterogeneous forms of rPSCs. How many different forms of rPSCs be generated? The multiple forms of rPSCs are not likely caused by multiple neuron classes. The body wall muscles in our preparation can only receive excitatory inputs from two classes of ventral nerve cord MNs, the A-MNs and VC-MNs. VC MNs regulate egg-laying that have less impact on body movement (Zhang et al., 2008). The remaining MNs (AS) only excite the dorsal muscles (Tolstenkov et al., 2018). Therefore, the observed excitatory rPSCs are mostly originated from A-MNs.

How are the mixed rPSCs generated by the A-MNs? One possibility is that each A-MN is capable of generating all types of rPSCs, and different types of rPSCs were stochastically generated. Anatomically, each ventral muscle sends 4–5 muscle arms to receive synaptic inputs from more than one ventral A-MN. Thus, another possibility is that the A-MN themselves have heterogeneous activity patterns, and the mixed rPSCs are generated from multiple A-MNs input onto the same muscle. Regardless, A-MNs are rigid oscillators with homogenous patterns, and they may innervate muscle with integrated activity patterns.

### Differential Regulation of Rhythmic Postsynaptic Currents by Ion Channels

The observation of different types of rPSCs in the isolated *C. elegans* backward motor circuit provides a rare opportunity for *in vivo* dissection of the molecular mechanism of motor rhythm. We identified the role of several Ca^2+^, Na^+^, and K^+^ channels in the rPSC pattern and motor rhythm (Supplementary Figures 4A,B). The Na^+^ leak channel NCA in depolarization of membrane potential, it was required for all types of rPSCs, consistent with their roles for basic resting membrane potential regulation (Lu et al., 2007; Xie et al., 2013).

However, other ion channels differentially regulated one or multiple classes of rPSCs. For example, the frequency and charge of the rPSCs could be modulated separately. The inward channels increase the activity of rPSCs, including the high-voltage-gated Ca^2+^ channels (VGCC), P/Q/N-type (UNC-2) and the L-type (EGL-19), and the Na^+^ leak channel NCA. Among them, UNC-2 preferentially promoted the tonic and phasic rPSCs, especially for the tonic-type. The involvement of the P/Q/N-VGCC in motor CPG has been reported in other vertebrate motor systems (Harris-Warrick, 2002; Grillner, 2003; Koch et al., 2013), which denotes the functional conservation of the regulation of these ion channels for motor rhythm.

EGL-19 specifically increased the activity of tonic rPSCs. Unlike UNC-2, which is expressed exclusively by neurons, EGL-19 is expressed by both neurons and muscles. Previously, we and others have shown that EGL-19 expressed by muscle cells is required for the action potential initiation (Gao and Zhen, 2011; Liu et al., 2011). We also implicated that EGL-19 expressed by neurons regulates the frequency of muscular action potentials (Gao and Zhen, 2011). A recent study showed that EGL-19 is also required for a sensory neuron AWA action potential firing (Liu et al., 2018). Here, the role of EGL-19 in rPSCs is of A-MN neuron origin. Its regulation of A-MN tonic rPSCs reveals multiple roles of *egl-19* in neurons and NMJs, from cellular and circuitry levels, respectively. Functional redundancy between different Ca^2+^ channels is well documented (Qian and Noebels, 2000; Takahashi et al., 2004a,b; Kaja et al., 2006). Our study similarly implicates potential functional redundancy between the UNC-2 and EGL-19 VGCC for tonic rPSCs in A-MNs, further demonstrated that the invertebrate nematode model with compact motor circuit is essential for motor rhythm dissection, specifically for molecular identity (Zhen and Samuel, 2015; Wen et al., 2018).

Three K^+^ channels, including BK/SLO-1, K_Na_/SLO-2, and Kv4/SHL-1, exhibited differential and partially overlapping inhibitory effects on the rPSCs. The BK/SLO-1 K^+^ channel was required for all types of rPSCs, but more important for tonic-type. K_Na_/SLO-2 K^+^ channel depressed the tonic and long-lasting rPSCs, and more critical for long-lasting-type. Kv4/SHL-1 K^+^ channel specifically decreased the activity of tonic rPSCs. These effects also implicate potential functional redundancy of the K^+^ channels.

The demonstration of spontaneous rhythmic PSCs in the simple backward motor circuit containing excitatory A-MNs and inhibitory D-MNs implicates that the existence of a functional attractor network in this circuit generates the finite rhythmic behavior. The identification of multiple components of these rPSCs from this circuit further indicates that this attractor is highly dynamic and probably be modulated by various factors. The ion channel configuration of this dynamic attractor was then partially addressed in this study. Additionally importantly, all channels that exhibit preferential regulation on the specific types of rPSCs also changed the reversal velocity. The dynamic rPSCs in wild-type and mutant animals that correlated with different velocities will help the attractor network simulation, which will shed light on our understanding in how fictive locomotion is generated in mammalian locomotion circuits.

### Optogenetic Isolation of a Neural Circuit

With a compact neural system and a conserved molecular repertoire, small animals serve as compact models to solve the similar challenges in locomotor rhythm faced by large animals. The isolated motor circuit—the spinal cord preparation of large animals—requires bathing in exogenously neuromodulators and young animals. Its activity could only be correlated with fixative locomotion. *C. elegans* motor circuit consists of a small number of neurons but a fundamental similarity in the circuit structure (Zhen and Samuel, 2015; Wen et al., 2018). Through genetic expression of a flavoprotein miniSOG, we have isolated a backward motor circuit in live animals and in the population (Gao et al., 2018). We demonstrated that this circuit exhibits intrinsic and oscillatory activity that is sufficient to drive rhythmic reversal in moving animals. This optogenetic strategy for circuit isolation in intact and behaving animals is of critical advantage to circuit studies.

### Limitations of the Study

We show here that three types of rPSCs constitute the A-MNs CPG rhythmic activity in *C. elegans*. These rPSCs occur either separately or simultaneously, without a clear sequential relationship. Whether these events are phase-locked or spontaneous and how the sequence of the events determines the reversal movements are largely puzzled. In the isolated preparations, transient single PSC bursts or multiphasic mPSCs were also observed. These PSC burst events are different from either the rPSCs or the mPSCs. What is the relationship between these events remains unknown and requires further investigation.

## Materials and Methods

### Constructs, Transgenic Arrays, and Strains

All *C. elegans* strains were cultured on the standard nematode growth medium (NGM) plates seeded with OP50 and maintained at 22°C (Brenner, 1974). Unless stated otherwise, the wild-type animal refers to the control transgenic strain. Strains that contain miniSOG transgene (*hpIs603*) were cultured in darkness on NGM plates. Only hermaphrodite worms were used for the experiments. Other genetic mutants used for constructing transgenic lines and compound mutants were obtained from the *Caenorhabditis Genetics Center* (*CGC*). All animals were backcrossed at least four times against N2 prior to analyses.

### MiniSOG-Based Neuron Ablation

For neuronal ablation constructs, miniSOG fused with an outer mitochondrial membrane tag TOMM20 (tomm20-miniSOG or mito-miniSOG) (Qi et al., 2012) was used. The constructs and sequence information referred to our previous description (Gao et al., 2018). The ablation of all members of premotor INs and B-MNs was performed using a homemade LED box, where the standard NGM culture plates located in without lid. Animals were exposed under 470 nm blue light (70 mW/cm^2^) for 40–45 min at 22°C. To monitor the specificity and efficacy of cell ablation, cytoplasmic RFP was coexpressed with miniSOG (tomm-20-miniSOG-SL2-RFP) in targeted neurons by the same promoter. Ablation was performed when animals were in the L2 stage.

### Behavioral Analyses

A single young adult hermaphrodite (12–18 h post-L4 stage), maintained on standard culture conditions, was transferred to a 60 mm imaging plate seeded with a thin layer of OP50. One min after the transfer, another one-min video of the crawling animal was recorded on a modified stereo microscope (Axio Zoom V16, Zeiss) with a digital camera (acA2500-60 um, Basler). Postimaging analyses utilized an in-house written MATLAB script. As we previously described, the central line was used to track. Images for velocity analysis from each animal were divided into 33 body segments. The midpoint was used to calculate the velocity and direction of movements between each frame.

Imaging plates were prepared as follows: a standard NGM plate was seeded with a thin layer of OP50 12–14 h before the experiment. Immediately before the transfer of worms, the OP50 lawn was spread evenly across the plate with a sterile bent glass rod. All images were captured with a 10X objective at 10 Hz.

### Fluorescence Microscopy

After the behavioral analysis, miniSOG animals were checked by fluorescence marker before and after LED illumination, respectively. They were mounted individually on agar pads to be examined for RFP signals; recordings from animals where RFP signals were absent were analyzed. Worms were immobilized with 2.5 mM levamisole (Sigma-Aldrich, United States) in M9 buffer. Fluorescence signals were captured from live worms using a Plan-Apochromatic 60X objective on a confocal microscope (FV3000, Olympus, Japan).

### *In vivo* Electrophysiology

Dissection and recording were carried out using protocols and solutions described in Gao and Zhen (2011), which were modified from Richmond and Jorgensen (1999) and Mellem et al. (2008). Briefly, 1- or 2-day-old hermaphrodite adults were glued (Histoacryl Blue, Braun) to a sylgard-coated cover glass covered with bath solution (Sylgard 184, Dowcorning) under a stereoscopic microscope (M50, Leica). After clearing the viscera by suction through a glass pipette, the cuticle flap was turned and gently glued down using WORMGLU (GluStitch Inc.) to expose the neuromuscular system. Body wall muscle cells were patched using 4–6 MΩ-resistant borosilicate pipettes (1B100F-4; World Precision Instruments). Pipettes were pulled by micropipette puller P-1000 (Sutter) and fire-polished by microforge MF-830 (Narishige). Membrane currents were collected in the whole-cell configuration by pulse software with an EPC9 amplifier (HEKA, Germany). Currents were recorded at a holding potential of –60 mV. Data were digitized at 10 kHz and filtered at 2.6 kHz. The pipette solution contains (in mM): K-gluconate 115; KCl 25; CaCl_2_ 0.1; MgCl_2_ 5; BAPTA 1; HEPES 10; Na_2_ATP 5; Na_2_GTP 0.5; cAMP 0.5; cGMP 0.5, pH 7.2 with KOH, ∼320 mOsm. cAMP and cGMP were included to maintain the activity and longevity of the preparation. The bath solution consists of (in mM): NaCl 150; KCl 5; CaCl_2_ 5; MgCl_2_ 1; glucose 10; sucrose 5; HEPES 15, pH 7.3 with NaOH, ∼330 mOsm. Chemicals and blockers were obtained from Sigma unless stated otherwise. Each animal was recorded for at least 5 min. Experiments were performed at room temperatures (20–22°C).

### Frequency Spectrum Analysis

Frequency spectrum analysis was used to transform the rhythmic PSC time waveform data into discrete frequency components by taking a fast Fourier transform (FFT) analysis. The Hamming window was used for the analysis in Clampfit 10.2 (Molecular Devices). The spectrum analysis showed that the dominant frequency of the phasic rPSCs is about 1–4 Hz and the effective frequency band is 1–10 Hz, whereas no similar dominant frequencies were observed in tonic and long-lasting rPSCs.

### Statistical Analysis

Two-tailed Student’s *t*-test was used to compare data sets. *p-*value < 0.05 was considered to be statistically significant; ^∗^, ^∗∗^, and ^∗∗∗^ denote *p* < 0.05, *p* < 0.01, *p* < 0.001, respectively. Graphing and subsequent analysis were performed using Igor Pro (WaveMetrics), Clampfit (Molecular Devices), ImageJ (National Institutes of Health), MATLAB (MathWorks, United States), GraphPad Prism 8 (GraphPad Software Inc., United States), and Excel (Microsoft, United States). For behavior analysis and electrophysiology, each recording trace was obtained from an individual animal (Supplementary Table 1). Unless specified otherwise, data were presented as the mean ± SEM.

## Data Availability Statement

The original contributions presented in the study are included in the article/Supplementary Material, further inquiries can be directed to the corresponding author/s.

## Author Contributions

BY and SG conceived the experiments and analyzed the data. BY and YW performed the experiments and analyzed the data. SG wrote the manuscript. All authors contributed to the article and approved the submitted version.

## Conflict of Interest

The authors declare that the research was conducted in the absence of any commercial or financial relationships that could be construed as a potential conflict of interest.

## Publisher’s Note

All claims expressed in this article are solely those of the authors and do not necessarily represent those of their affiliated organizations, or those of the publisher, the editors and the reviewers. Any product that may be evaluated in this article, or claim that may be made by its manufacturer, is not guaranteed or endorsed by the publisher.

## Supplementary Material

Supplementary Table 1 The original data points and statistics of all figures.
