# Supplementary material for: Motor Rhythm Dissection From the Backward Circuit in C. elegans
Source: Front Mol Neurosci. 2022 Mar 16;15:845733. doi: 10.3389/fnmol.2022.845733 (PMC8966088; doi:10.3389/fnmol.2022.845733)
Supplement: Supplementary file 2 [file Data_Sheet_1.pdf]

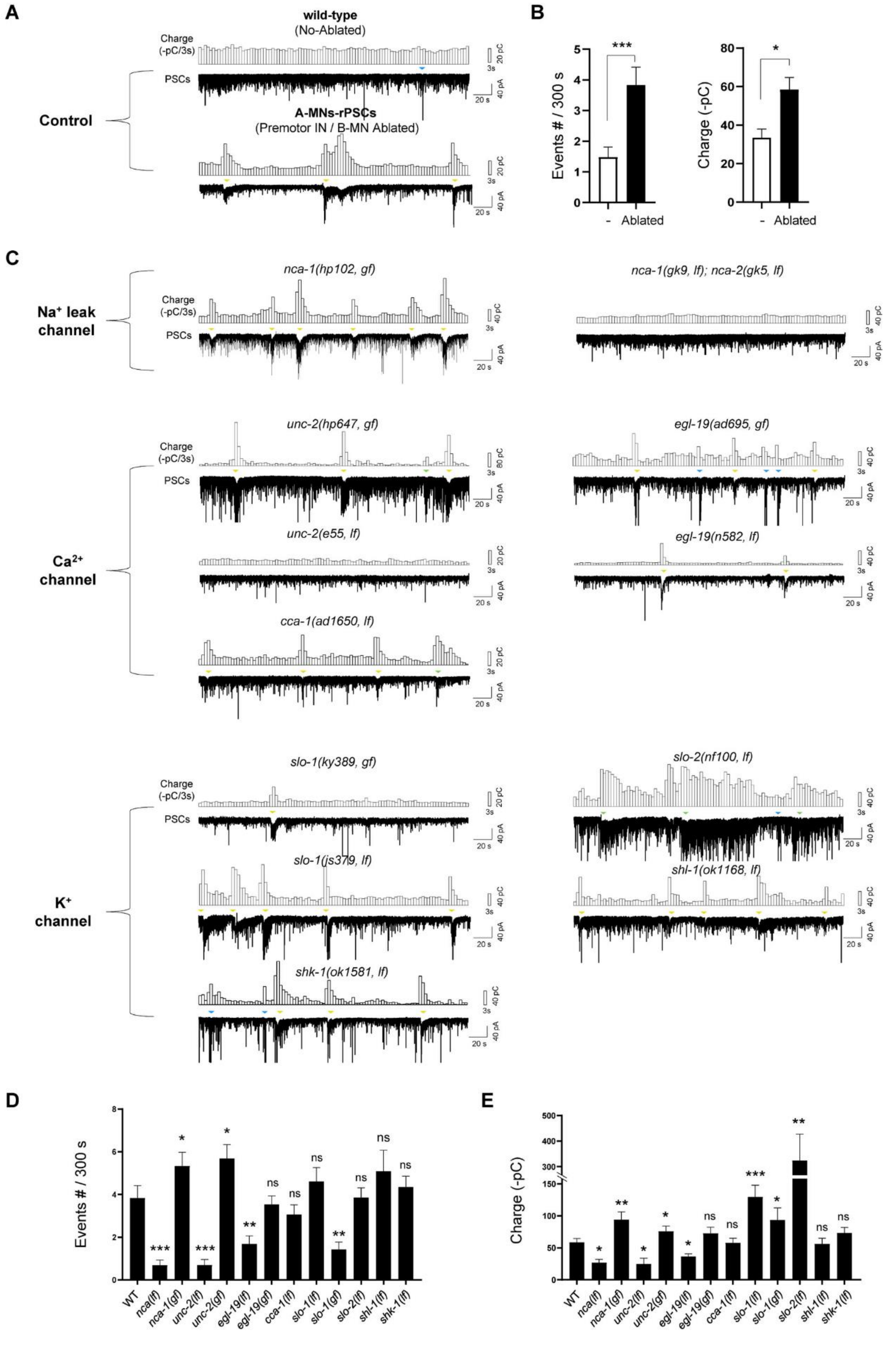

Figure S1. Robust rhythmic activities require multiple ion channels

(**A**) Representative spontaneous PSCs trace and continue charge frequency (–pC/3s) in

wild type animal without (Control) the ablation of premotor INs and B-MNs. (**B**) Quantification of the total rPSCs frequency and charge with and without (-) the ablation. n ≥ 19 animals in each group. (**C**) Representative rPSCs traces and continue charge frequency analysis (–pC/3s) from different genotypes. (**D, E**) Quantification of the total rPSCs frequency and charge in all genotypes. All data are expressed as means ± SEM. The Student's *t*-test was used. Statistical significance is indicated as follows: **P* < 0.05, ***P* < 0.01, ****P* < 0.001 in comparison with that as denoted.

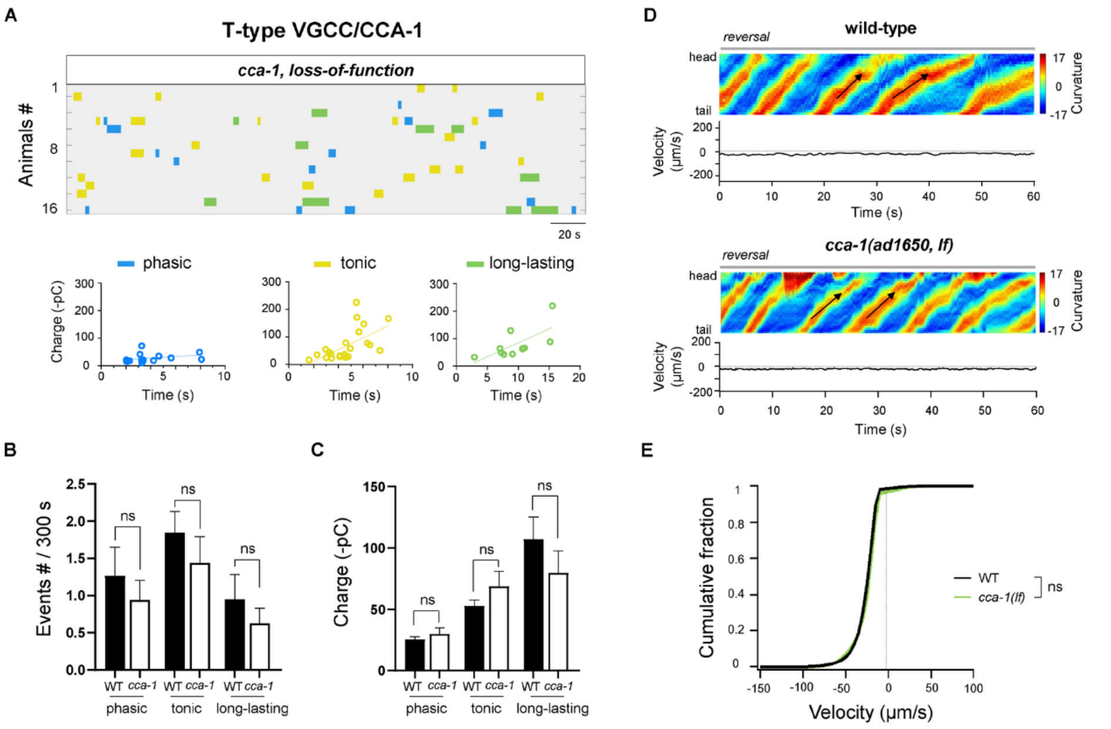

Figure S2. T-VGCC/CCA-1 is not required for rPSCs

(**A**) Color-map and charge efficiency (linear fitting) of all rPSC events of ablated *cca-1(ad1650; lf)* mutant animals. Blue dots denote phasic-type events, yellow dots denote tonic-type events, and green dots denote long lasting-type events. (**B, C**) Quantification of the frequency and charge of different rPSCs in *cca-1(ad1650; lf)* mutant. n ≥ 16 animals in each group. (**D**) Representative curvature kymogram (*up*) and instantaneous

velocity (*bottom*) of free-behaving animals of *cca-1(ad1650; lf)* mutants, which exhibit normal reversal, as posterior to anterior propagating body bends (downward arrows). (**E**) Distribution of instantaneous velocity of wild type, *cca-1(ad1650; lf)* mutant animals. The velocity fraction is overlapped in wild type and *cca-1(ad1650; lf)* mutant animals. n ≥ 10 in each group. ns $P > 0.05$ against ablated wild type by the Kolmogorov-Smirnov test. All data are expressed as means ± SEM. The Student's *t*-test was used (B, C). Statistical significance is indicated as follows: ns, not significant in comparison with that as denoted.

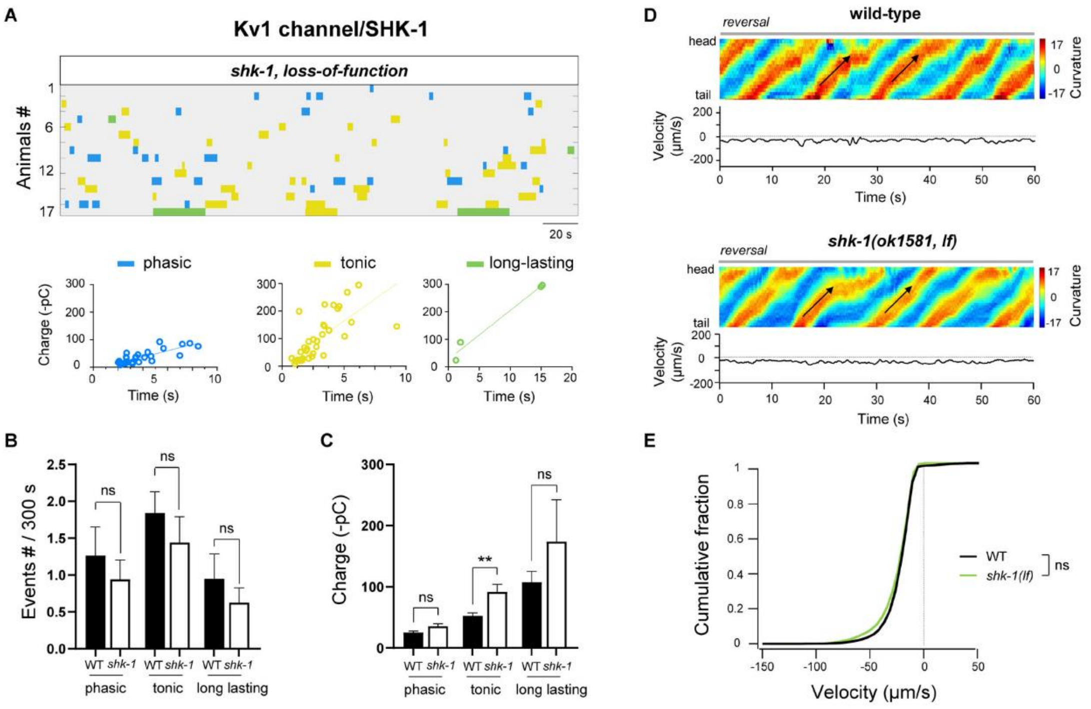

Figure S3. Reversal is not affected in ablated shk-1(lf) mutant

(**A**) Color-map and charge efficiency (linear fitting) of all rPSC events of ablated *shk-1(ok1581; lf)* mutant animals. Blue dots denote phasic-type events, yellow dots denote tonic-type events, and green dots denote long lasting-type events. (**B, C**) Quantification of the frequency and charge of different rPSCs in respective genotypes. n ≥ 17 animals

in each group. (**D**) Representative curvature kymogram (*up*) and instantaneous velocity (*bottom*) of free-behaving animals of respective genotypes. *shk-1(ok1581; lf)* mutant animals exhibit a comparable reversal with wild type animals. (**E**) Distribution of instantaneous velocity of wild type, *shk-1(ok1581; lf)* mutant animals. n ≥ 10 in each group. ns $P > 0.05$ against ablated wild type by the Kolmogorov-Smirnov test. All data are expressed as means ± SEM. The Student's *t*-test was used (B, C). Statistical significance is indicated as follows: ns, not significant, $^{**}P < 0.01$ in comparison with that as denoted.

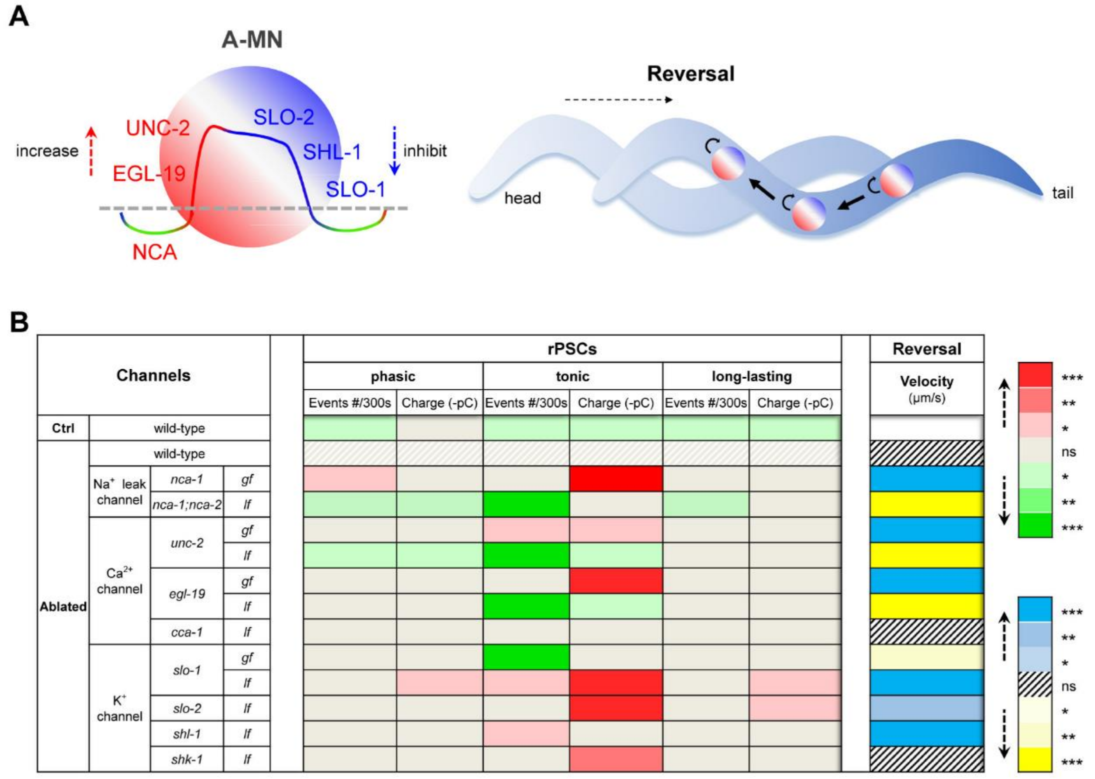

Figure S4. Summary of the effects of different ion channels on rPSCs and behavior

(**A**) A working model that ion channels regulate distinct parts of the A-MNs oscillating activity, from which NCA/UNC-2/EGL-19 Na^+/Ca^{2+} channels promote the activity, SLO-2/SHL-1/SLO-1 K^+ channels inhibit the activity. (**B**) Summary table to show the

significant differences on rPSCs and reversal velocity in different genotypes. Statistical significance is indicated as follows: ns, not significant, $*P < 0.05$, $**P < 0.01$, $***P < 0.001$ in comparison with ablated wild type animals (shaded grids). Red bars denote increase, green bars denote decrease, tawny bars mean not significant.
